# High-efficiency grating couplers for vertical coupling in thin-film silicon nitride technology

**DOI:** 10.1038/s41598-026-44998-0

**Published:** 2026-04-20

**Authors:** Francesca di Croce, Valerio Vitali, Thalía Domínguez Bucio, Anna Pennoni, Hao Liu, Ilaria Cristiani, Frederic Gardes, Cosimo Lacava, Periklis Petropoulos

**Affiliations:** 1 Electrical, Computer and Biomedical Engineering Department, University of Pavia, Pavia, 27100 Italy; 2 Optoelectronics Research Centre, University of Southampton, Southampton, SO17 1BJ UK

**Keywords:** Engineering, Materials science, Optics and photonics, Physics

## Abstract

Silicon nitride has become increasingly prominent in the integrated photonics research community during the last two decades, with an ever-growing number of demonstrations reported for different applications. In particular, thin-film silicon nitride platforms are attracting increasing interest thanks to the possibility of achieving ultra-low propagation loss due to their weak mode-field confinement. However, the realization of efficient grating couplers in the thin-film silicon nitride platforms is extremely challenging due to the poor grating scattering strength. In this work, we propose and experimentally demonstrate a grating coupler for perfectly-vertical fiber-to-chip efficient coupling to thin-film silicon nitride waveguides. The device consists of a silicon-rich silicon nitride apodized grating on top of a 150 nm-thick silicon nitride waveguide. Unlike most previously demonstrated bi-layer grating couplers, which typically employ an amorphous silicon layer to enhance the grating efficiency, the use of a top silicon-rich silicon nitride grating offers improved fabrication tolerances and gives an additional degree of freedom in the design of efficient grating couplers. A record coupling efficiency of -1.78 dB at 1550 nm was experimentally measured without employing any back-reflector or index-matching fluid, demonstrating the device’s potential for efficient and fabrication-tolerant fiber-to-chip coupling on thin-film silicon nitride platforms.

## Introduction

Over the last twenty years, photonic integrated circuits (PICs) have revolutionized a wide range of research fields and applications, including on-chip sensing^1^, high-performance computing^2^, all-optical signal processing^3,4^, and quantum technologies^5,6^. Across a wide range of applications, PICs could outperform microelectronic-based approaches in terms of operational speed and energy efficiency. However, reducing insertion losses, including both waveguide propagation losses and fiber-to-chip coupling losses, remains a critical challenge that must be addressed to enable the large-scale deployment of PICs.

In this context, integrated photonics based on the silicon nitride (SiN) platform has attracted considerable attention in recent years, mainly due to its low propagation losses, wide optical transparency range, and compatibility with standard silicon photonics fabrication techniques^7,8^. In addition to the possibility of achieving low waveguide propagation losses, several approaches for the realization of efficient grating couplers (GCs) for SiN waveguides with a thickness $\ge$ 300 nm have been demonstrated. For such thicknesses, the grating scattering strength allows achieving a reasonable matching between the field scattered by the GC and the optical fiber field profile by implementing proper GC apodization techniques^9,10^. Additionally, the GC directionality can be increased by incorporating a bottom reflector^9,11–13^ or an additional GC level^10,14–18^.

Recently, research interest has also focused on developing ultra-low-loss photonic platforms based on thin-film silicon nitride (TFSN) waveguides, which consist of high aspect-ratio optical waveguides with thicknesses in the range $\approx$ 40 - 200 nm^19^. Due to the minimal mode-field confinement in the waveguide core and the excellent material properties of stochiometric TFSN films deposited by low-pressure chemical vapor deposition (LPCVD) processes, extremely low propagation losses in the range $\approx$ 0.1 - 10 dB/m were demonstrated^20–24^. At present, TFSN platforms are being actively studied for various on-chip functionalities such as sub-Hertz fundamental linewidth Brillouin lasers^25^, distributed Bragg reflector lasers based on hybrid SiN waveguides coated with rare-earth-doped materials^26^, high quality-factor ring resonators for frequency comb generation^27^, true time delay lines for microwave photonics^28^, and arrayed waveguide grating spectrometers for astronomical applications^29^, to report a few examples.

While the extremely low mode-field confinement in the waveguide core allows achieving low propagation losses, it poses some critical challenges to the development of high-efficiency GCs. The relatively small refractive index difference between the core and cladding leads to weak grating diffraction strength, which, in turn, reduces the GC directionality and mode-matching, ultimately limiting the overall coupling efficiency (CE). As for the case of thicker SiN platforms, solutions based on bi-layer GCs^10,19,24,30^ or the use of bottom back-reflectors^19,30^, like metal mirrors or distributed Bragg reflectors (DBRs), have been reported to enhance the CE, with the best results in the telecommunications C-band summarized in Table 1. However, the use of back-reflectors typically increases the fabrication complexity and may result in poor fabrication tolerances^19^. In bi-layer GCs, the integration of an additional high-index material, typically silicon (Si) or amorphous Si, can be employed to increase the GC directionality and, hence, the CE. Nevertheless, the exploitation of an additional GC layer with a relatively high refractive index commonly results in poor fabrication tolerances, particularly for apodized GCs^24^. Moreover, the implementation of such schemes may require chemical mechanical polishing (CMP) for surface planarization, as well as precise layer alignment when the two GC levels are implemented through separate etching steps^10^.

**Table 1:** Comparison of TFSN GCs in the telecommunications C-band reported in the literature. CE$_{sim}$: numerically simulated CE; CE$_{meas}$: experimentally measured CE (NA = not available).

Reference	SiN [nm]	GC configuration and notes	CE$_{sim}$ [dB]	CE$_{meas}$ [dB]
^30^	40	Bi-layer Si-on-SiN apodized GC, with DBR	−0.4	NA
^24^	100	Bi-layer Si-on-SiN uniform GC	−3.8	−5
^19^	125	Uniform Si GC embedded in a SiN layer, with DBR	−1.3	−2.22
^19^	125	Apodized Si GC embedded in a SiN layer, with DBR	−0.28	−1.84
^10^	150	Dual-level (dual-etch) Si-on-SiN GC	−0.75	NA
This work	150	SRSN-on-SiN apodized GC for vertical coupling	−1.37	−1.78

In this work, we discuss the design, fabrication, and characterization of a silicon-rich silicon nitride (SRSN)-on-SiN GC for light coupling in a 150 nm-thick SiN waveguide, and we report a record CE of −1.78 dB at 1550 nm for the transverse electric (TE) polarization. This result is achieved without any back-reflector and for vertical fiber-to-chip coupling, unlike the GCs reported in the literature for TFSN platforms (see Table 1), which were designed for a specific, non-vertical fiber angle. The table presents the thickness of the SiN layer, the GC configuration used, the best CE found in the simulations, and the measured CE. While a few papers report simulated couplers with higher CE^10,19,30^, this work shows the highest measured CE, demonstrating the potential of this structure. High-efficiency perfectly-vertical GCs are ideal candidates for PICs because they can reduce packaging complexity and ease wafer-scale testing^31^.

Our approach involves using an SRSN grating above the SiN waveguide, where only the grating is etched, leaving the underlying SiN waveguide intact. This platform design has been explored in other work that numerically demonstrates the effectiveness of using a uniform amorphous silicon (aSi) grating on top of a SiN waveguide, which eliminates the alignment challenges common in bi-layer grating couplers^32^. While prior studies have shown the theoretical benefits of structures with uniform GC, our work introduces a fully etched apodized grating designed to improve the overlap between the diffracted field and the optical fiber mode, enhancing the coupling efficiency. We demonstrate the effectiveness of this approach both theoretically and experimentally, achieving the highest measured CE among those reported in Table 1, validating the advantage of our design.

Finally, the use of SRSN introduces an additional degree of freedom in the design of GCs, thanks to the possibility of precisely tuning its refractive index by adjusting the material composition during the deposition process. This flexibility makes the approach suitable not only for different SiN thicknesses, but also for other material platforms^8,33^. Moreover, as we numerically demonstrate, employing SRSN as the top GC material allows for improved fabrication tolerances compared to the case in which Si is used.

## Grating coupler layout and numerical simulations

**Fig. 1:**
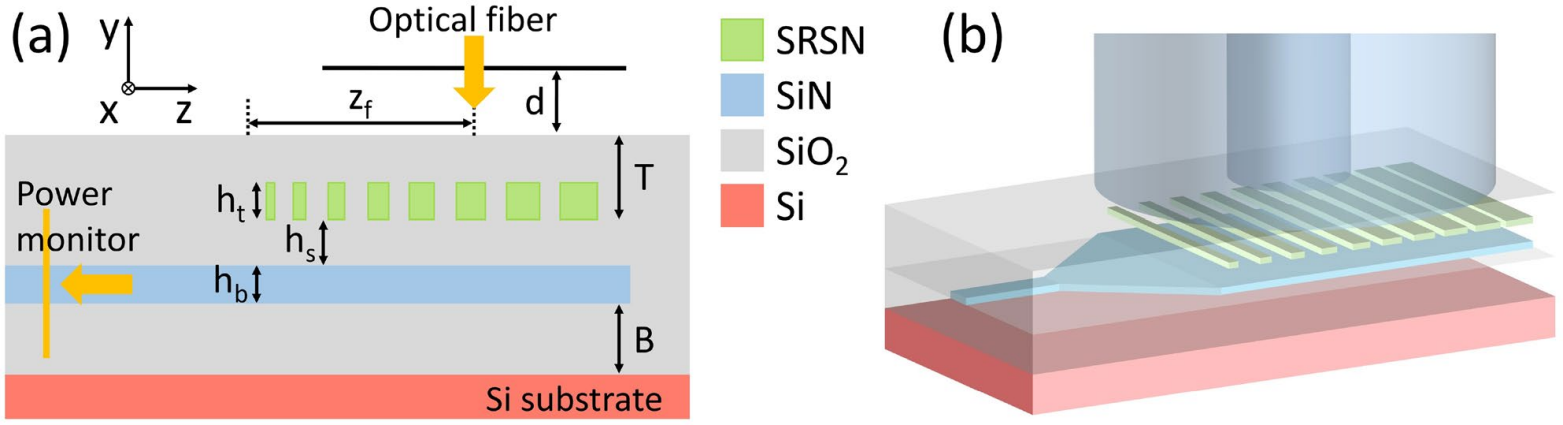
(**a**) 2D and (**b**) 3D layouts of the demonstrated GC.

In standard uniform grating couplers (UGCs), light is extracted from a guided mode by means of a periodic modulation of the effective refractive index along the propagation direction. This modulation is obtained by etching trenches of fixed length and depth in the waveguide, with a constant period $\Lambda$. A key parameter describing each scattering element is the grating duty cycle (DC), defined as the ratio between the tooth length $L_o$ (i.e., the un-etched section) and the total period $\Lambda$. The effective refractive index of each grating period can be written as

1 $$\begin{aligned} n_{eff} = DC \cdot n_{o}+ (1-DC) \cdot n_{e}, \end{aligned}$$

where $n_o$ and $n_e$ are the effective refractive indices of the un-etched and etched waveguide sections, respectively. The periodic alternation between these two regions produces a longitudinal variation of $n_{eff}$, which enables diffraction of the guided mode into free space. The direction of the diffracted beam is governed by the first-order Bragg condition, which relates the grating period to the coupling wavelength $\lambda _c$ and to the diffraction angle $\theta$ in air:

2 $$\begin{aligned} \Lambda = \frac{\lambda _{c} }{n_{eff} - sin \theta } . \end{aligned}$$

For a uniform GC, where both DC and $\Lambda$ are constant, the Bragg condition is satisfied identically by all scattering elements, resulting in a well-defined diffraction angle. However, such a uniform configuration generally leads to a poor overlap between the exponentially decaying field radiated by the grating and the Gaussian-like mode profile of a single-mode optical fiber, ultimately limiting the achievable coupling efficiency. To mitigate this limitation, linear apodization of the duty cycle is commonly employed. In an apodized grating coupler, the duty cycle is gradually varied along the propagation direction z, allowing the scattering strength of each grating period to be tailored. In particular, weaker scattering near the beginning of the GC and progressively stronger scattering toward the end enable a more uniform power extraction along the grating length, thereby improving the overlap between the radiated field and the fiber mode. A linear apodization of the duty cycle can be expressed as:

3 $$\begin{aligned} DC(z) = DC_i + R_{DC} \cdot z, \end{aligned}$$

where $DC_{i}$ is the initial duty cycle of the first scattering element and $R_{DC}$ is the apodization rate. While this approach effectively controls the local radiation strength, it also introduces an intrinsic limitation: varying *DC* along the grating directly modifies the local effective refractive index $n_{eff}$ (z), as evident from (1). As a consequence, if the grating period $\Lambda$ is kept constant, the Bragg condition of (2) can no longer be simultaneously satisfied along the entire grating. Instead, phase matching occurs only at a specific position where the local value of $n_{eff}$(z) matches the design condition, while the remaining scattering elements operate under a phase mismatch. This effect leads to a progressive deterioration of the constructive interference among the radiated fields and a reduction of the overall grating directionality. To overcome this fundamental trade-off between apodization and phase matching, a more general design strategy consists in simultaneously apodizing both the duty cycle and the grating period. By properly adjusting the period of each scattering element according to the local effective refractive index, the Bragg condition can be approximately satisfied at every position along the grating:

4 $$\begin{aligned} \Lambda (z) = \Lambda _i - R_{\Lambda } \cdot z. \end{aligned}$$

where $\Lambda _{i}$ is the initial period of the first scattering element and $R_{\Lambda }$ is the apodization rate. In practice, this is commonly implemented by introducing a linear chirp of the grating period with opposite sign with respect to the duty cycle apodization. This complementary apodization preserves phase matching while keeping the benefits of a tailored radiation profile, resulting in enhanced fiber–grating mode overlap and higher coupling efficiency. This theoretical approach, which utilizes a tailored radiation profile to optimize the fiber–grating mode overlap and enhance coupling efficiency, is implemented in the proposed device, where its two-dimensional (2D) and three-dimensional (3D) GC layouts are shown in Fig. 1 (a) and (b), respectively. The device consists of a TFSN waveguide layer (bottom layer, thickness $h_b$ = 150 nm, refractive index = 2.01 at 1550 nm) deposited on a buried silicon dioxide (SiO$_2$) layer (thickness B = 3070 nm, determined by the starting wafer used for fabrication). A SRSN GC layer (top layer, thickness $h_t$, refractive index = 2.54 at 1550 nm) is separated by a SiO$_2$ buffer (thickness $h_s$) from the TFSN waveguide. Waveguides and linear tapers connecting different devices are defined in the bottom TFSN layer, while apodized GCs are patterned exclusively in the top SRSN layer. This design approach allows to significantly relaxing fabrication tolerances associated with possible mask misalignment, which are critical in multi-etch GC designs^34^. In this work, a simultaneous apodization of both the period $\Lambda$ and DC was applied to the SRSN grating coupler to minimize the mode mismatch between the grating and the optical fiber. A SiO$_2$ cladding layer (TOX, thickness *T*) was deposited after the SRSN layer etching. The GC was designed for a standard single-mode optical fiber (SMF-28), with an outer diameter of $125 \, \mu \text {m}$ and a mode field diameter (MFD) of $10.4 \, \mu \text {m}$ at 1550 nm, which was placed perpendicularly to the chip surface. In numerical simulations, the optical fiber was modeled as a Gaussian beam embedded in the air region above the SiO$_2$ cladding layer, positioned at a fixed distance d = $0.5 \, \mu \text {m}$ in the y-direction and at an offset position $z_f$ from the center of the beam to the start of the GC in the z-direction.

**Table 2:** Optimal dimensions (period $\Lambda$ and tooth width $L_o$) obtained from the optimization of the apodized SRSN-on-SiN grating coupler with SRSN thickness $h_t$ = 150 nm.

*No*	$\Lambda$ [$\mu m$]	$L_o$ [$\mu m$]	*No*	$\Lambda$ [$\mu m$]	$L_o$ [$\mu m$]
1	1.024	0.205	11	0.955	0.580
2	1.017	0.246	12	0.949	0.613
3	1.010	0.287	13	0.942	0.645
4	1.003	0.326	14	0.935	0.677
5	0.996	0.365	15	0.929	0.708
6	0.989	0.403	16	0.929	0.708
7	0.982	0.440	17	0.929	0.708
8	0.975	0.476	18	0.929	0.708
9	0.969	0.511	19	0.929	0.708
10	0.962	0.546	20	0.929	0.708

The layout of the proposed GC is designed to simultaneously increase the grating directionality and reduce the mode mismatch between the grating-scattered field profile and the Gaussian-like optical fiber mode. The enhanced directionality can be attributed to the fact that adding the top grating layer further“breaks”the symmetry of the GC compared to single-level grating approaches. This asymmetry allows the GC to be designed to maximize upward diffraction while suppressing downward scattering toward the substrate^34^.

A minimum feature size of 205 nm was considered, which is compatible with our deep-UV (DUV) lithography capabilities. The optimization of the GC parameters to maximize the CE was achieved by performing full vectorial 2D-FDTD numerical simulations using Ansys Lumerical FDTD (©2025 Copyright ANSYS, Inc), considering the GC as an in-coupling device, i.e. coupling light from the optical fiber into the TFSN waveguide by means of the GC (see Fig. 1 (a)). A frequency-domain power monitor along the TFSN waveguide was employed to measure the power in the fundamental TE mode, from which the CE was derived. The free GC parameters {$h_s$, $h_t$, *T*, $DC_i$, $R_{DC}$, $\Lambda _i$, $R_{\Lambda }$, $z_f$} were optimized to maximize the CE at 1550 nm for the fundamental TE mode by using the Ansys Lumerical FDTD particle swarm optimization (PSO) algorithm. The GC dimensions for each period of the optimized structure are listed in Table 2. In the optimization process, the fiber angle was fixed at 0$^\circ$ to find the optimized parameters for perfectly-vertical coupling.

**Fig. 2:**
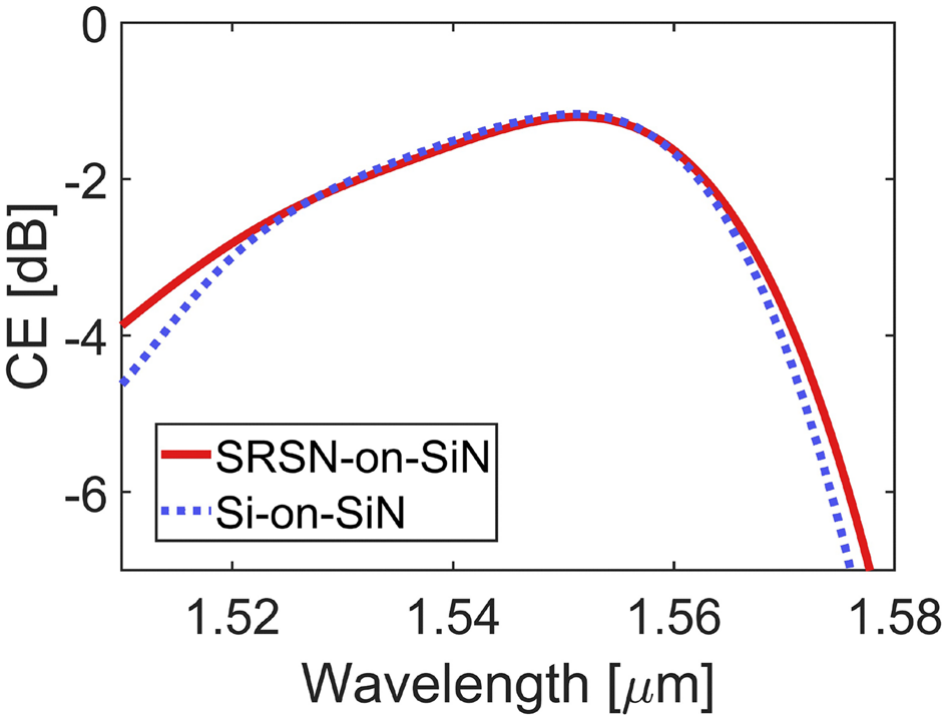
Numerically simulated CE versus wavelength for the SRSN-on-SiN (red solid line) and Si-on-SiN (blue dashed line) GCs. SRSN-on-SiN parameters: $$h_s$$ = 180 nm, $$h_t$$ = 150 nm, T = 1390 nm, $$DC_i$$ = 0.2, $$R_{DC}$$ = $$0.041 \, \mu \text {m}$$$$^{-1}$$, $$\Lambda _i$$ = 1024 nm, $$R_{\Lambda }$$ = 0.00693 m$$^{-1}$$, $$z_f$$ = $$5.74 \, \mu \text {m}$$. Si-on-SiN parameters: $$h_s$$ = 190 nm, $$h_t$$ = 50 nm, T = 1370 nm, $$DC_i$$ = 0.2, $$R_{DC}$$ = $$0.0465 \, \mu \text {m}$$$$^{-1}$$, $$\Lambda _i$$ = 1024 nm, $$R_{\Lambda }$$ = 0.0067 m$$^{-1}$$, $$z_f$$ = $$5.9 \, \mu \text {m}$$.

**Fig. 3:**
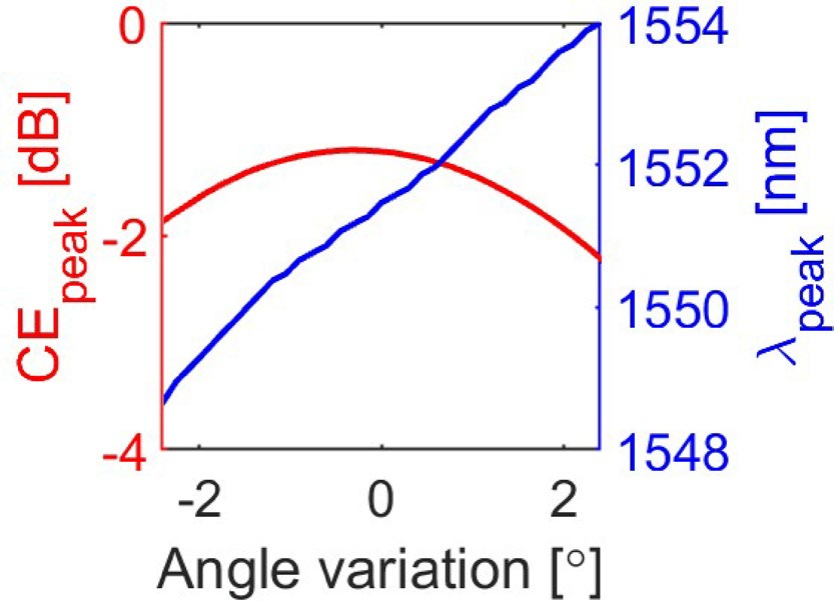
Effect of angle variation on the peak coupling efficiency ($CE_{peak}$) and peak coupling wavelength ($\lambda _{peak}$).

The use of two linear apodizations with opposite chirping signs for the *DC* and period allows to define all the grating trenches and teeth by considering only four parameters ($DC_i$, $R_{DC}$, $\Lambda _i$, $R_{\Lambda }$) instead of optimizing each GC period and *DC* independently. This approach enables the simultaneous evaluation of a wide range of parameters and multiple GC configurations using the PSO algorithm, including variations in the separation between the two layers and the top GC thickness. Fig. 2 shows the CE as a function of wavelength for the best SRSN-on-SiN GC (red solid line) resulting from the PSO algorithm. A peak CE and a 1dB bandwidth (BW) equal to −1.21 dB and 35.9 nm were found, respectively.

The effect of angle variation on the peak CE ($CE_{peak}$) and peak coupling wavelength ($\lambda _{peak}$) was then evaluated and is shown in Fig. 3. For comparison, the same simulation campaign was repeated using a Si (refractive index = 3.48 at 1550 nm) top GC instead of the SRSN one, and the resulting CE versus wavelength for the best design is shown in Fig. 2 (blue dashed line). As can be seen, the two approaches yield similar performance in terms of peak CE and 1 dB BW. The parameter values that maximize the CE for the two designs are listed in the caption of Fig. 2. The main difference between the two parameter sets lies in the top GC thickness $h_t$, whereas all other parameters have similar values. As expected, using SRSN as the top GC material results in a thicker optimal layer ($h_t$ = 150 nm) compared to when Si is used ($h_t$ = 50 nm) due to the lower SRSN refractive index.

**Fig. 4:**
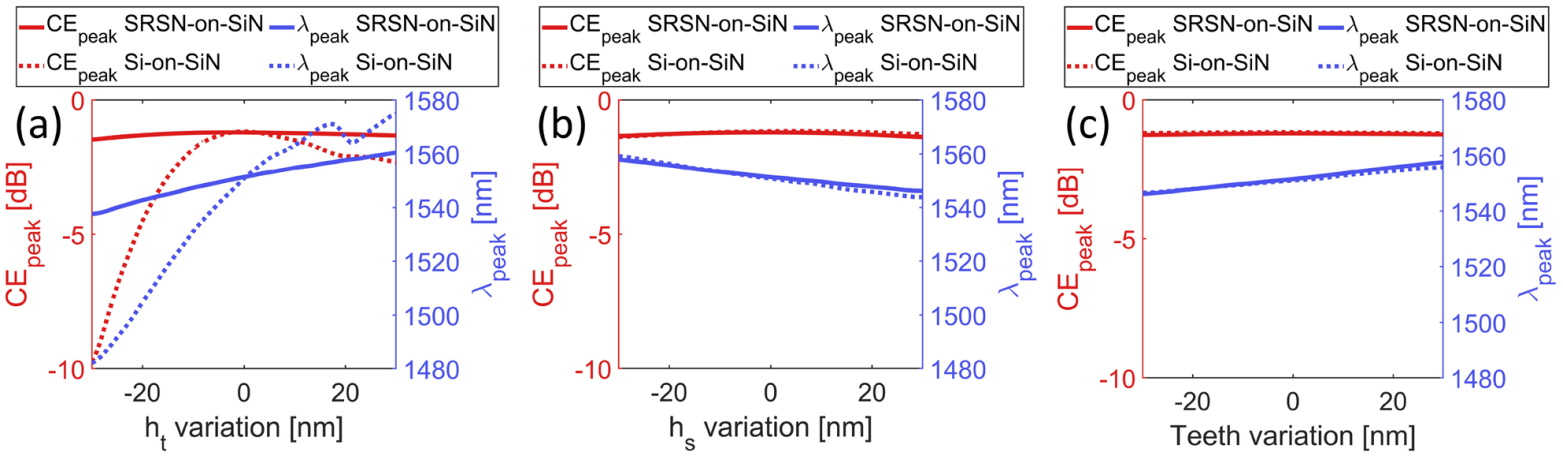
Design sensitivity to critical fabrication parameters: dependence of $CE_{peak}$ (red lines) and $\lambda _{peak}$ (blue lines) on variations of (**a**) the top layer thickness $h_t$, (**b**) SiO$_2$ buffer thickness $h_s$, and (**c**) grating teeth dimensions.

The sensitivity of the two designs to fabrication errors was then evaluated. Specifically, Fig. 4 (a), (b), and (c) show the impact of deviations from the nominal values of the top layer thickness $h_t$, SiO$_2$ buffer thickness $h_s$, and grating teeth dimensions on the peak CE ($CE_{peak}$) and peak coupling wavelength ($\lambda _{peak}$), respectively. While both designs show relatively good and comparable fabrication tolerances for the last two parameters, the use of SRSN leads to a more robust design against variation of the top layer thickness $h_t$ compared to the use of Si, both in terms of $CE_{peak}$ and $\lambda _{peak}$.

## Device fabrication

**Fig. 5:**
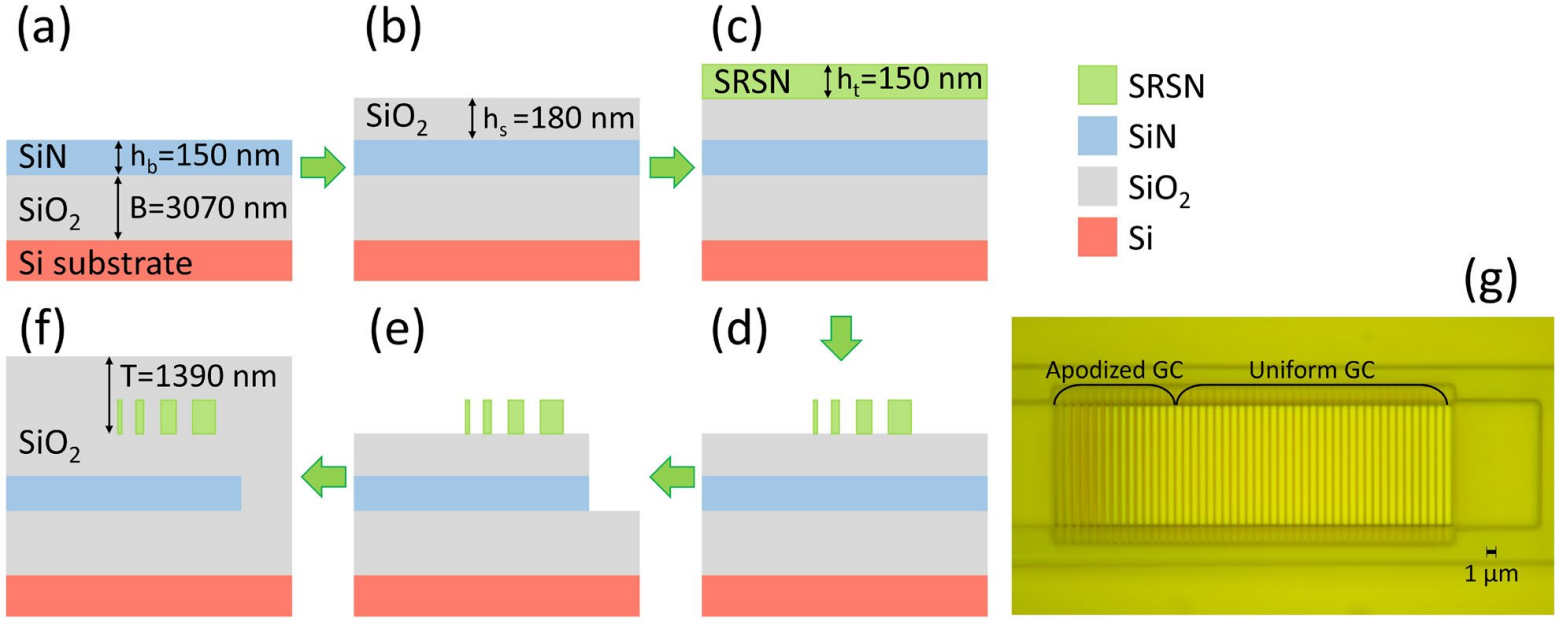
Fabrication steps for the bi-layer SRSN-on-SiN GCs: PECVD-based depositions of (**a**) a 150 nm SiN layer, (**b**) a 180 nm SiO_2_ buffer layer and (**c**) a 150 nm SRSN layer to define the bi-layer GC stack starting from a 200 mm silicon wafer with a 3070 nm thermal SiO_2_ layer; (**d**) top SRSN GC patterning and etching; (**e**) SiN waveguide level etching; (**f**) deposition of a 1390 nm thick PECVD SiO_2_ layer. (**g**) Microscope image of a fabricated GC.

The SRSN-on-SiN GC was fabricated using a 200 mm silicon wafer as a starting substrate with a 3070 nm thermal SiO_2_ layer. The stack comprising a 150 nm SiN layer, a 180 nm SiO_2_ buffer layer, and a 150 nm SRSN layer was deposited by plasma-enhanced chemical vapor deposition (PECVD) (Fig. 5 (a)-(c)). These processes used an N$_2$+SiH$_4$ chemistry for the SiN and SRSN layers^35^, and a SiH$_4$+N$_2$O+N$_2$ chemistry for the SiO_2_ layer. The SRSN top layer was then exposed by means of a 248 nm DUV lithography scanner using a 680 nm KrF-M91Y positive tone resist. The pattern was then transferred to the wafer through an inductively coupled plasma (ICP) etching process using an SF$_6$+C$_4$F$_8$ chemistry (Fig. 5 (d)). At this stage, the top SRSN layer was completely removed from the waveguide area, apart from the GC regions. Once the resist was removed using an O$_2$ plasma and a wet RCA1 cleaning process, the SiN waveguides were patterned using DUV lithography. The waveguides were then etched by using an ICP etching process to remove the SiO_2_ buffer layer and then fully etch the SiN waveguide layer using an SF$_6$+C$_4$F$_8$ chemistry (Fig. 5 (e)). The fabrication was finalized by cladding the devices with a 1390 nm thick PECVD SiO_2_ layer deposited with the same chemistry used for the SiO_2_ buffer layer (Fig. 5 (f)). Fig. 5 (g) shows a microscope image of a fabricated GC. As can be seen, at the end of the apodized GC section, consisting of 15 periods, a uniform GC section (tooth width: 708 nm; trench width: 221 nm) is added to ensure that all the optical power in the waveguide is scattered, thereby minimizing back-reflection at the end of the grating.

**Fig. 6:**
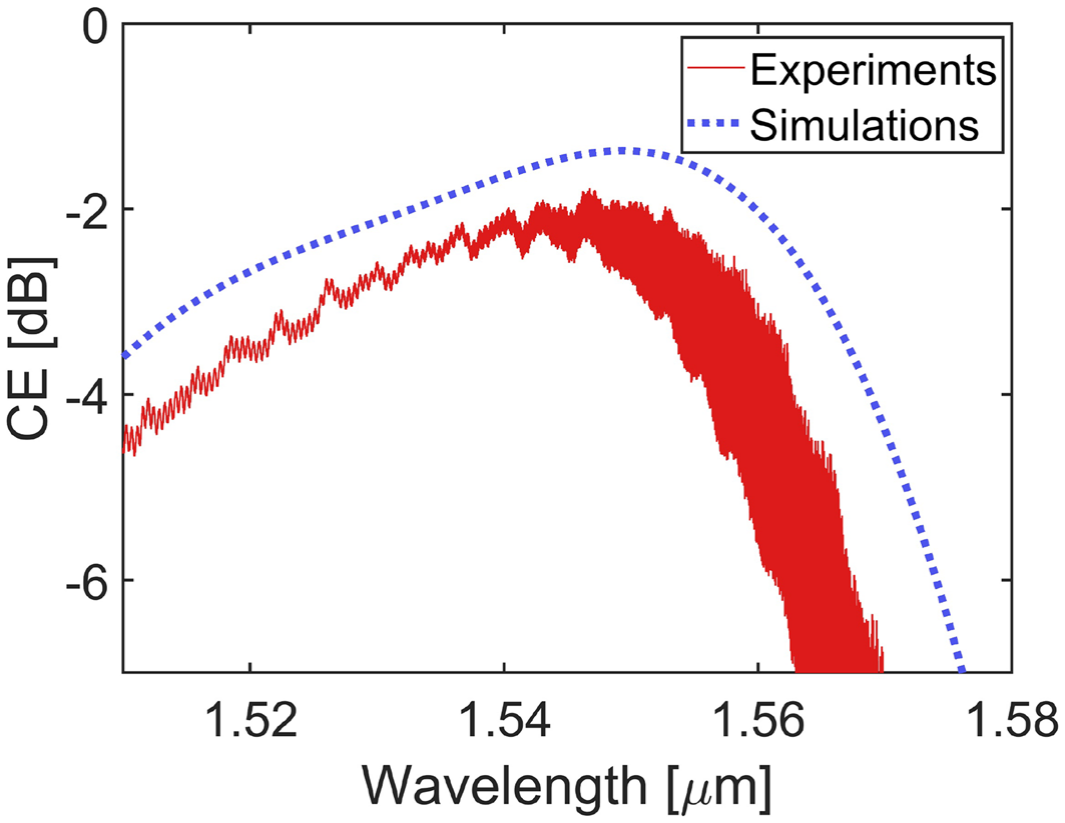
3D-numerically simulated (blue dashed line) and experimentally measured (red solid line) CE versus wavelength for the fabricated GC.

The width of the GC was set equal to $14 \, \mu \text {m}$. Fig. 6 shows the CE as a function of wavelength resulting from a full-vectorial 3D-FDTD simulation that was performed on the optimized SRSN-on-SiN GC to account for the GC width (blue dashed line). A CE peak at 1550 nm equal to −1.37 dB (0.16 dB lower than the CE value resulting from the 2D simulation) and a 1 dB BW of 37 nm were numerically found. To experimentally characterize the performance of the GCs, we fabricated structures featuring a pair of GCs connected by a 1 cm long straight SiN waveguide (width: $2.5 \, \mu \text {m}$; thickness: 150 nm). The GCs were connected to the SiN waveguides using $400 \, \mu \text {m}$ long linear tapers. To separate the contributions of the GC coupling losses from the waveguide propagation losses, spiral waveguides with various lengths were fabricated to carry out cut-back measurements.

## Results

The grating CE was obtained by subtracting the waveguide propagation loss from the measured fibre-to-fibre transmission and dividing by two (thus assuming that the input and output coupling losses could be considered equal, thanks to the reciprocity theorem). The experimentally measured CE as a function of wavelength for the SRSN-on-SiN GC is shown in Fig. 6 (solid red line). A peak value for the grating CE equal to −1.78 dB at a wavelength of $\approx$ 1547 nm and a 1 dB BW of $\approx$ 31 nm were experimentally measured.

The discrepancy between the simulated CE of −1.37 dB and the experimentally derived value of −1.78 dB may be attributed to small fabrication imperfections, such as variations in material thicknesses or the uniformity of the deposited TOX layer, as well as to topographical effects derived from the absence of post-process planarization. Additional simulations including a SiO$_2$ GC on top of the TOX to approximate the grating-like shaped real surface indicate a CE reduction of about 0.3 dB, considering the worst-case scenario.

Dedicated simulations of the back-reflection for the proposed design have been performed. The results show a back-reflection of approximately 1% at 1550 nm, which corresponds to the central operating wavelength of the device. As expected, the back-reflection increases for wavelengths far from the central wavelength. In particular, at longer wavelengths, a significant increase in retro-reflection was observed.

In the experimental grating transmission spectrum, a parasitic amplitude ripple is observed, which could be caused by residual grating reflectivity. The ripple period (approximately 0.07 nm) corresponds to a cavity of length 1.08 cm, which corresponds to the waveguide length, suggesting the presence of parasitic reflections between the two grating structures.

## Discussion

In conclusion, we have reported the design, fabrication, and experimental characterization of a GC for efficient fiber-to-chip vertical coupling to TFSN waveguides. The demonstrated device comprises an SRSN GC on top of a TFSN layer, where only the optical waveguides are defined, thereby minimizing fabrication imperfections related to mask misalignment for the patterning of the two layers, which are critical for GCs with two or multiple etching steps. The duty cycle and the period of the SRSN GC are both linearly apodized, with opposite chirping signs. The use of a top SRSN GC allows achieving more relaxed fabrication tolerances thanks to the larger GC dimensions and lower refractive index contrast. An experimental CE of −1.78 dB and a 1 dB BW of 31 nm were demonstrated in the telecommunications C-band without the use of any back-reflector, highlighting the potential of the device for efficient fiber-to-chip coupling into low-loss TFSN platforms. We believe that, thanks to its versatility and the additional degree of freedom given by the use of SRSN as a GC material, the design approach presented in this work could also be employed for the realization of fabrication-tolerant and high-efficiency GCs in other integrated photonic platforms.

## Methods

All finite-difference time-domain (FDTD) numerical simulations were conducted using FDTD Solutions™(Lumerical Inc.). The two-dimensional (2D) computational domain was defined with a width of $36.5 \, \mu \text {m}$ and a height of $7 \, \mu \text {m}$. The refractive index of SiO$_2$ was obtained from the data reported by Palik, yielding a value of n$_{SiO_2}$ =1.44 at a wavelength of 1550 nm. For the SiN and SRSN materials, the refractive indices used in the simulations were n$_{SiN}$ =2.01 and n$_{SRSN}$ =2.54, respectively, at 1550 nm. The simulation mesh was defined using a conformal meshing method implemented in Lumerical, with the highest accuracy setting (value of 8). Additionally, a locally refined mesh with a minimum feature size of 10 nm was applied within the grating coupler (GC) region. For each grating design, a total of 21 periods was considered. The optical power diffracted upward by the grating coupler, used to evaluate its directionality, was calculated using a frequency-domain power monitor with 500 frequency sampling points over the wavelength range of 1.45 to 1.65 $\mu \text {m}$. The optical fiber source was modeled as a Gaussian beam with a mode field diameter (MFD) of $10.4 \, \mu \text {m}$, positioned in the air region above the top SiO$_2$ cladding. The beam was oriented parallel to the vertical direction, with the electric field polarized along the x-axis, enabling coupling to the fundamental TE$_{00}$ mode of the waveguide. A frequency-domain power monitor placed along the TFSN waveguide, $12.5 \, \mu \text {m}$ from the start of the grating coupler, was used to calculate the optical power coupled into the fundamental TE mode of the waveguide and consequently to determine the coupling efficiency (CE).

The experimental characterization was conducted by using a vertical coupling setup with vertically-aligned polarization-maintaining (PM) SMF-28 optical fibers, properly placed to couple light into the fundamental TE waveguide mode. A PM tunable laser was employed as the optical source, while a power meter was used to measure the power at the output of the device under test. The measurements were performed without the use of any index-matching fluid between the optical fibers and the chip TOX.

## Data Availability

The data underlying the results presented in this paper are available from the corresponding author upon reasonable request.
